# Heart Transplantation: Immunological Challenges Revisited

**DOI:** 10.2174/011573403X362826250619112705

**Published:** 2025-06-25

**Authors:** Sara Assadiasl, Sepehr Safdel, Mahsa Gheitasi, Mohammad Hossein Nicknam

**Affiliations:** 1 Molecular Immunology Research Center, Tehran University of Medical Sciences, Tehran, Iran;; 2 Iranian Tissue Bank and Research Center, Tehran University of Medical Science, Tehran, Iran;; 3 Department of Immunology, School of Medicine, Kerman University of Medical Sciences, Kerman, Iran;; 4 Department of Immunology, School of Medicine, Tehran University of Medical Sciences, Tehran, Iran

**Keywords:** Heart transplantation, allografts, graft rejection, immunologic surveillance, biomarkers, immunotherapy

## Abstract

**Introduction:**

Immunologic responses to cardiac allografts initiate before transplantation during brain-dead organ procurement and might persist for years after transplantation, culminating in chronic allograft dysfunction. Despite remarkable advances in post-transplant care and immunosuppressive agents, acute cellular and antibody-mediated rejections as well as chronic allograft vasculopathy significantly affect cardiac allograft and patient survival.

**Methods:**

Herein, recent findings of the molecular mechanisms involved in the inflammatory responses before and after heart transplantation, including brain death donor inflammation, acute cellular rejection, antibody-mediated rejection, and chronic allograft dysfunction, have been summarized, along with novel therapeutic approaches for their treatment. Finally, recent developments in prognostic and diagnostic biomarkers for immunological complications have been provided, with an overview of the most promising biomarkers to date.

**Results and Discussion:**

Due to the recent developments in the description of molecular mechanisms involved in the immunopathogenesis of cardiac allograft rejection, some immune cells, proinflammatory cytokines, and adhesion molecules have been proposed as therapeutic targets for the prevention or treatment of alloimmune responses. In addition, several molecules derived from graft tissue or immune cells, *e.g.* natriuretic peptides, cardiac troponins, exosomal products, microRNAs, and donor-derived cell-free DNA, have been suggested as potential biomarkers for the prediction or diagnosis of cardiac transplant rejection.

**Conclusion:**

Considering the need to design non-invasive, low-cost tests for early diagnosis of post-transplant complications and convenient follow-up of the cardiac transplant recipients, peripheral blood biomarkers could be appropriate candidates for this purpose.

## 1 INTRODUCTION

Heart transplantation is the standard treatment for patients with end-stage heart failure refractory to medical treatments, including ischemic and non-ischemic cardiomyopathies, and valvular heart diseases. In recent years, the rate of heart transplantation worldwide has reached 1.5 per million population with one-year and five-year survival of approximately 90% and 80%, respectively [1]. In addition to various obstacles to successful heart transplantation *e.g.* surgical, hemodynamic, and infectious complications, the immune responses before, during, and after transplantation affect allograft survival. Alloimmune responses might sustain insidiously for years leading to chronic allograft vasculopathy, for which there is no definite treatment yet. The requirement for non-invasive, low-cost, and rapid diagnostic tests for acute and chronic rejection is the other unsolved problem in cardiac transplantation as currently, the gold standard of diagnosis is endomyocardial biopsy accompanied by significant complications [2]. Therefore, immunological aspects of heart transplantation and new developments in the pathogenesis, diagnosis, and treatment of immune responses need further consideration. In the present review, the latest findings of molecular mechanisms involved in immunological complications, along with potential therapeutic targets and diagnostic biomarkers, have been discussed, with a brief review of the current knowledge of heart transplant rejection.

Two independent literature searches were performed using Scopus, PubMed, and Google scholar search engines. Keywords including “heart transplant”, “cardiac transplant”, “cardiac allograft”, “brain-dead donor”, “acute rejection”, “chronic rejection”, “desensitization”, “antibody-mediated rejection”, “biomarker”, and “outcome” were searched in two-keyword combinations. In “title search”, 12706 articles were detected, from which, 1145 articles were selected for “abstract search”, and 323 articles were considered eligible for “text reading” among which 161 articles were included in the results section.

## 2 IMMUNE RESPONSES TO HEART ALLOGRAFT

### 2.1 Brain-dead Donor Inflammation

Inflammatory responses in brain-dead (BD) organs before transplantation have been demonstrated with experimental and human studies. It is currently believed that ischemia/reperfusion and preliminary disorders of the BD donor cause tissue injury and release of danger-associated molecular patterns (DAMPs), which result in inflammation before transplantation [3]. One of the first experiments reported increased expression of intercellular adhesion molecule-1 (ICAM-1), vascular adhesion molecule-1 (VCAM-1), interleukin (IL)-1β, and IL-6 in heart tissue of BD rats compared to the control animals, indicating an endothelial activation that induced leukocytes extravasation [4]. A similar study showed upregulated expression of IL-1β, monocyte chemoattractant protein-1 (MCP-1), tumor necrosis factor-alpha (TNF-α), and transforming growth factor beta (TGF-β) genes in cardiac tissue from BD rats. Moreover, two weeks after transplantation, mononuclear cell (MNC) infiltration was four-fold more than in the heart transplant from living donors. In the long-term follow-up, the mRNA level of these genes reduced to the baseline in the latter group, whereas it remained high in the former. The histological assessment also demonstrated widespread fibrosis in the cardiac graft from BD rats, which was restricted to minor areas in the living donor group. Infiltration of CD45+ cells, T cell receptor (TCR), and macrophages in the recipients from BD donors was also significantly higher than in the living graft recipients [5, 6]. Likewise, a human study comparing the plasma cytokine level of BD patients with the septic cases and ICU-admitted controls demonstrated comparable levels of IL-6, IL-8, TNF-α, and interferon-γ (IFN-γ) in BD and septic patients that were significantly higher than in ICU-admitted non-septic individuals [7]. Complement activation and deposition of C3 components have also been observed in the vascular endothelium and surrounding myocytes of BD mice models. Besides, C3 deficiency was associated with reduced tissue inflammation [8]. A similar experiment showed increased expression of C3, TNF-α, ICAM-1, Chemokine (C-C motif) ligand 5 (CCL5), and T cell infiltration in BD organs [9]. These and other findings indicate the initiation of immune responses in heart tissue before transplantation and allorecognition.

### 2.2 Acute Cellular Rejection

There is a growing body of evidence indicating that the increased expression of proinflammatory cytokines and chemokines due to BD donors, prolonged ischemia time, and infections might lead to early graft dysfunction [10]. A genetic assay showed Chemokine (C-X-C motif) ligand 9 (CXCL9), CXCL10, CXCL11, and CXCR3 expression in human cardiac allografts, which was absent in normal heart tissue. Persistent expression of the CXCL10 and CXCL11 was associated with cardiac vasculopathy; moreover, CXCL9 and CXCL10 expression was shown to result in leukocyte infiltration and acute rejection [11]. Similarly, endomyocardial biopsy (EMB) specimens from 30 heart transplant recipients revealed increased expression of the CXCL10, CXCL9, CXCL11, CCL5, CXCR3, and CCR5 but not MCP-1 and IL-8 in grades 2 and 3 rejection [12]. Comparing chemokine expression in EMBs from 24 patients during early (first 3 months) and late (9^th^ to the 12^th^ month) episodes of acute rejection showed an upregulated expression of CXCL10, CXCL9, CCL5, CXCR3, and CCR5 in the late *versus* early biopsies but there was no significant difference between rejection grades [13]. Later, one study examining CD4+ T-cell subsets in peripheral blood and EMB of cardiac transplant recipients demonstrated increased expression of T-bet, IFN-γ, RORγt, IL-17, IL-23, and FoxP3 in the endomyocardium of patients with acute rejection, which was accompanied by elevated proportion of Th1, Th17, and FoxP3+CD4+ T cells in peripheral blood [14]. Another study demonstrated a lower Treg to endothelial progenitor cell ratio (Treg/EPC ratio) early after transplantation in recipients with acute rejection [15].

### 2.3 Antibody-mediated Rejection

Both preformed and *de novo* antibodies might contribute to alloimmune responses and affect graft survival. These antibodies could be auto- or alloantibodies directed to HLA or non-HLA antigens. The non-HLA targets of antibodies could be tissue-specific (exclusively expressed in the heart) or ubiquitous antigens expressed in several tissues [16]. Studies have shown that HLA-I antigen crosslinking by antibodies stimulated the upregulation of P-selectin on endothelium. P-selectin-induced monocyte adhesion was further enhanced by secondary interactions of IgG with Fc-gamma receptors (FcγRs), which resulted in leukocyte recruitment to the allograft [17]. Moreover, histological evaluation of EMBs from cardiac transplant recipients with pre- or post-transplant donor-specific antibodies (DSA) demonstrated a significantly increased ratio of CD8/CD4 T cells in allograft suggesting a crucial role for cytotoxic T cells in the pathogenesis of antibody-mediated rejection (AMR) [18]. There was also a significant correlation between AMR and the proportion of circulating recent thymic emigrant CD4+ T cells. T cell receptor excision circle levels as well as thymus volume and density were higher in these patients. It is worth noting that all subjects received antithymocyte globulin therapy before heart transplantation [19].

Endothelium-directed antibodies can also activate the complement system that exacerbates inflammatory responses and causes further tissue injuries. A C1q solid-phase assay that specifically detected complement-fixing DSA in cardiac transplants reported a higher incidence of acute cellular and antibody-mediated rejection in the recipients with DSA than those without DSA. In addition, the patients with C1q+ DSA had more AMR episodes than the group with C1q- DSA. Patients with *de novo* DSA had lower survival rates than patients with no DSA, but recipients with complement-fixing DSA and non-complement-fixing DSA showed comparable survival rates [20]. Furthermore, some single nucleotide polymorphisms (SNPs) in donors *e.g.* interleukin-4 receptor subunitα (p.Ile75Val-IL4Rα), which is involved in B cell biology, were associated with the development of AMR in recipients [21].

As mentioned before, besides anti-HLA antibodies, antibodies targeting non-HLA antigens such as vimentin, beta-tubulin, lamin A/C, and apolipoprotein L2 may play a role in AMR since a cohort study demonstrated that elevated reactivity to autoantigens was associated with a higher probability of AMR and graft failure in heart transplant recipients, particularly in DSA-positive patients [22]. A pediatric case, retransplanted after developing CAV, was reported to have high levels of non-HLA antibodies directed to angiotensin II receptor type 1 (AT1R) and donor-specific MHC class I chain-related gene A (MICA) antigens, resulting in the AMR and accelerated CAV of the second transplant [23]. Moreover, certain infections are supposed to induce or accelerate AMR. A cohort study of 47 heart transplant recipients, 24 with AMR and 23 without AMR episodes, demonstrated a significant association between AMR and viral infections *i.e.* cytomegalovirus (CMV) and Epstein-Barr virus (EBV) early after transplantation [24]. In addition, latent CMV infection might be involved in the endothelial injury of cardiac allograft since endothelial function, defined as flow-mediated dilation, was significantly impaired in patients with CMV replication in leukocytes compared to the patients without infection [25]. There were also reports of developing AMR with or without cellular rejection in heart transplant recipients with previous COVID-19 infection or vaccination [26].

### 2.4 Chronic Allograft Dysfunction / Chronic Vasculopathy of Cardiac Allograft

Chronic rejection of heart transplants or cardiac allograft vasculopathy (CAV) is the main cause of late death in heart transplant recipients. Given that there is no definitive treatment for chronic rejection, the only alternative is retransplantation, which usually occurs approximately 7 years after the first transplant [27]. Although the exact mechanism has not yet been understood, it appears that the vascular inflammation and endothelial dysfunction induced by ischemia-reperfusion injury, recurrent episodes of acute rejection, and circulating DSAs lead to injury-repair cycles causing luminal stenosis, tissue fibrosis, and thrombotic events in the allograft [28]. There is evidence of systemic inflammation presented with elevated CRP levels in heart transplant recipients showing progressive CAV compared to those with a non-progressive course of the disease [29]. In addition, the gene and protein expression of toll-like receptor 4 (TLR4) and its downstream molecules including B7-1, IL-12, and TNF-alpha were upregulated in circulating monocytes of cardiac transplant recipients with allograft endothelial dysfunction [30]. Moreover, mRNA expressions of matrix metalloproteinase (MMP)-2 and MMP-9 in donor spleen lymphocytes and in biopsy samples one week after transplantation were associated with an increased risk of developing myocardial fibrosis and coronary vasculopathy in the 12^th^-month biopsies [31]. Furthermore, pediatric recipients diagnosed with CAV had higher levels of circulating IL-6, VCAM, ICAM, and thrombomodulin compared to control subjects [32]. IL-3 may also contribute to allograft fibrosis in a basophil-dependent manner; moreover, basophil-derived IL-6 could be involved in fibrosis progression [33]. Single-cell RNA sequencing analysis showed that allograft-infiltrating macrophages had a proinflammatory and glycolytic phenotype with enhanced activation of MEK/ERK signaling; accordingly, trametinib, an FDA-approved MEK1/2 inhibitor, ameliorated chronic cardiac allograft rejection in an experimental model [34]. Furthermore, it was found that in contrast to IFN-γ-/- mice that were protected from CAV, T-bet-/- recipients developed an accelerated allograft rejection accompanied by early severe vascular inflammation, IL-17-producing CD4 T cells infiltration, and increased production of IL-4, IL-5, IL-10, IL-13, IL-6, IL-12, and IL-17. Neutralization of IL-17 inhibited allograft rejection and vasculopathy in T-bet-/- mice, suggesting a role for Th17 cells in accelerated cardiac vasculopathy [35]. Likewise, the tissue expression of Th17-related genes, including Foxp3, IL-17, and IL-23, was increased in a murine model of cardiac allograft vasculopathy compared with Th1-related genes [36]. Since miR-155 plays a role in the differentiation and stability of the Th17 cells, miR-155-/- mice exhibited resistance to cardiac rejection. Notably, this effect was reversed by recombinant IL-17A [37]. Moreover, dysregulation of purinergic receptor-7 (P2X7R) signaling that controls Th17 and Th1 generation and differentiation resulted in an abnormal Th17 generation and poor cardiac allograft outcomes in patients with P2X7R mutations [38]. Despite these findings, IL-17 blockers such as Secukinumab and Ixekizumab have not been tested for the prevention or treatment of CAV in humans.

There is also a growing body of evidence demonstrating a significant association between alloantibodies and developing CAV. One study showed that recipients diagnosed with CAV developed DSA and auto-Abs to collagen-V (Col-V), with increased frequencies of Th17 cells specific for Col-V and K-α1-Tubulin [39]. In addition, an experimental study reported a correlation between CAV and DSA titers; besides, transcriptomic analysis of microdissected allograft arteries identified the Notch ligand Dll4 as the most elevated transcript in CAV, associated with the high titers of DSA [40]. The implication of natural killer (NK) cells has also been shown in developing CAV as in an experiment depletion of NK cells with anti-NK1.1 mAbs significantly reduced the severity of DSA-induced CAV. This pathway might explain the C4d-negative chronic antibody-mediated rejection in humans [41]. The role of NK cells in the progression of CAV was attributed to IL-6 production by FcγR-activated NK cells [42].

High-mobility group box 1 (HMGB1) seems to be involved in CAV *via* dendritic cell (DC) activation because HMGB1-specific neutralizing mAbs substantially ameliorated CAV and tissue fibrosis. It significantly reduced DC number, T cell infiltration, and IL-17A/IFN-γ production in allograft and spleen [43]. Additionally, HMGB1-neutralizing antibodies have been found to attenuate allograft fibrosis by decreasing the transformation of fibroblasts to myofibroblasts and inhibiting TGF-β1 production. Besides, recombinant HMGB1 stimulation promoted *in-vitro* release of TGF-β1 from cardiac fibroblasts and macrophages [44]. Similarly, higher connective tissue growth factor (CTGF) protein expression was observed in the cardiomyocytes of a CAV model [45]. Another study demonstrated the advantages of decorin, an extracellular matrix protein that inhibits TGF-β function, in heart transplantation since gene transfer of decorin into allografts significantly attenuated interstitial fibrosis and cardiac hypertrophy without affecting T-cells’ alloresponsiveness [46]. In line with this, GeoMx digital spatial profiling used to analyze arterial areas of interest from CAV+DSA+ rejected cardiac allografts showed increased TGF-β-regulated transcripts and growth factors-associated proteins in correlation with modules enriched for proliferation/repair [47]. These findings suggest a significant role for TGF in the pathogenesis of cardiac allograft rejection; however, there is not yet any report of TGF inhibition in transplant recipients, and regarding the immunomodulatory role of TGF-β, the safety of such therapeutics is still debated.

## 3 THERAPEUTIC STRATEGIES

Considering the above-mentioned findings, some therapeutic strategies have been invented to inhibit or reduce the intensity of immunological damage in cardiac allografts. Herein, recent advances in the prevention and treatment of brain-dead donor inflammation, acute cellular rejection, AMR, and CAV have been summarized.

### 3.1 Pretransplant Management of BD Heart

So far, treatment strategies such as intensive hemodynamic management, administration of vasopressin, and hormone therapy have been suggested to reduce tissue injury in BD hearts [48]; however, the outcomes are not satisfying. A comparison between two groups of BD pigs receiving either methylprednisolone (MP) or placebo demonstrated apoptosis and upregulated expression of IL-1, 6, 10, ICAM-1/2, TNF-α and VCAM-1 in the right ventricle of both groups with less intensity in MP-treated pigs [49]. Likewise, a clinical trial including 4 groups of BD donors treated with tri-iodothyronine (T3), methylprednisolone, both, or placebo showed elevated serum levels of IL-1, IL-6, TNF-α, CRP, and procalcitonin in all donors with no considerable difference between groups [50]. Therefore, novel approaches are required to reduce inflammation and improve the quality of BD organs. For instance, vagus nerve stimulation was applied in a rat model of BD that improved cardiac function and inhibited TNF production [51]. Another experiment demonstrated reduced levels of IL-1, IL-6, TNF-α, and IFN-γ in BD mice treated with thymoglobulin. Heart from these donors showed less MNC infiltration and graft quality comparable to the organs from living donors [52]. In line with this, another experiment showed that 45 minutes after a single infusion of thymoglobulin to the mice, IL-6 and IL-2 receptor expression was significantly downregulated in the myocardium [53]. Hypertonic saline, as a volume expander, improved hemodynamic stability and ejection fraction in BD rats; moreover, the expression of anti-apoptotic molecules increased whereas TNF and VCAM expression decreased [54]. 17β-estradiol has also been tested to improve the quality of cardiac allograft in female rats. Administration of this hormone immediately after BD induction or three hours later resulted in significantly lower troponin-I levels. Besides, caspase-3, ICAM-1, leukocyte infiltration, and hemorrhage were reduced in graft while B-cell lymphoma 2 (Bcl-2) and endothelial nitric oxide synthase (eNOS) levels showed upregulation [55]. Furthermore, treating aortic rings from BD rats with mesenchymal stem cell-derived conditioned medium reduced the expression of myeloperoxidase (MPO), caspase-3, -8, and -9, as well as VCAM-1 and ICAM-1, in the aortas compared to saline-treated animals [56]. Recently, in an experiment, either tacrolimus or placebo was administered to the pigs before BD induction. BD led to upregulated Bax/Bcl2 and IL-6/IL-10 ratios and enhanced VCAM-1 expression in the myocardial tissue of the placebo group, which was significantly mitigated in the tacrolimus group. Neutrophil infiltration in the ventricles was also inhibited. As a result, tacrolimus reduced pulmonary vascular resistance, prevented RV-arterial decoupling, and improved the function of the BD heart [57]. These reports suggest the administration of immunosuppressive agents, including drugs used for pretransplant induction, *e.g.* thymoglobulin, or maintenance therapy, *e.g.* tacrolimus, to the BD donors before harvest. However, interventions such as mesenchymal stem cell-derived exosomes might be less feasible for human BD donors due to lower availability and high costs.

### 3.2 Acute Rejection

Regarding the implication of T cells in acute rejection, they have been targeted to improve allograft survival. A clinical study including 15 patients who received thymoglobulin infusion *via* coronary sinus intraoperatively and immediately after organ procurement and 15 patients who received traditional thymoglobulin infusion after implantation reported lower inotropic therapy doses and fewer acute rejection episodes in the former group [58]. In another study, daclizumab, a monoclonal anti-IL-2 receptor antibody, was administered to cardiac transplant recipients that significantly reduced the rate of acute rejection in recipients with one-locus human leukocyte antigen (HLA)-DR mismatch. Nonetheless, in two-loci DR mismatch recipients, there was no difference between daclizumab-treated and control patients [59]. A similar study showed a higher incidence of acute rejection, antibody production, and graft failure in heart transplant recipients who received no or fewer doses of daclizumab compared to those who took higher doses. This study also demonstrated a comparable function of CD25+FOXP3+ Treg cells in daclizumab-treated patients and control recipients, which, considering the importance of IL-2 in the generation and function of Treg cells, was of significance [60]. Janus Kinase (JAK) inhibition has also been considered in the prevention and treatment of allograft rejection [61]; JAK2/STAT3 signaling inhibition with AG490 reduced the risk of acute rejection in mice cardiac transplant, probably *via* suppressing the Th17/IL-17 axis [62]. Similarly, ruxolitinib, a JAK1/2 inhibitor, prolonged cardiac allograft survival by targeting the signaling pathway of IFNGR1. Of note, ruxolitinib showed higher efficacy than IL-2RA mAbs such as daclizumab because it did not affect Treg activation, which is IL-2-dependent [63]. These reports suggest novel immunosuppressive agents *e.g.* anti-IL-2 receptor antibodies and JAK inhibitors, besides standard treatment, to treat patients at higher risk of acute rejection; nonetheless, their safety need to be evaluated in large cohorts.

In an experimental study, infusion of bone marrow-derived mesenchymal stem cells (MSC) to recipient mice the day before heart transplantation combined with low dose tacrolimus resulted in a significant increase in survival rate and decreased risk of acute rejection. Pathological assessment demonstrated lower infiltration of proinflammatory cells in allograft and reduced IFN-γ expression in MSC/TAC therapy [64]. Moreover, IL-35 gene-modified adipose-derived MSCs produced exosomes with immunosuppressive activity that prolonged cardiac allograft survival in murine models and increased the Treg ratio in blood circulation [65]. Although MSC therapy has been considered in the treatment of numerous inflammatory disorders, its superiority over conventional treatments of acute rejection needs to be established. Extracorporeal photopheresis (ECP), defined as treating white blood cells with 8-methoxy psoralen and subsequent irradiation with ultraviolet A light, has also shown encouraging results in the prevention and treatment of acute rejection in experimental models [66]. Additionally, some plant-derived traditional extracts such as Rho and Matrine have been proposed to improve the anti-inflammatory effects of the standard treatments mainly through inhibition of DC maturation [67, 68].

There are other experimental studies with unknown relevance in human subjects that might be considered in future studies. For example, according to the increased metabolism of glucose in cardiac macrophages during acute rejection and the role of hypoxia-inducible factor 1 subunit alpha (HIF1A) in the regulation of glycolysis, HIF1A inhibition in a murine model of acute rejection reduced the antigen presentation and proinflammatory cytokine secretion in macrophages and mitigated the infiltration of CD4+ and CD8+ T cells [69]. Nuclear receptor subfamily 4 Group A member 1 (Nr4A1) has also been found to increase during cardiac allograft rejection, particularly in graft-infiltrating CD4+T cells. Since Nr4A1 is supposed to be involved in CD4+ T differential apoptosis, administration of its agonist cytosporone B to mice resulted in non-Treg cell apoptosis and Treg cell differentiation, thus reducing acute rejection risk and increasing graft survival [70]. Another experiment showed that CXCR3 blockade significantly inhibited the alloreactive T cell function and attenuated acute cardiac allograft rejection [71]. Later, a CXCL10+Gbp2+ cluster of cardiac fibroblasts was detected in mice heart allograft with strong interferon responsiveness and high expression of chemokines and HLA molecules that were probably involved in the recruitment and activation of immune cells, further suggesting CXCR3 inhibition to prolong allograft survival [72]. Finally, it appears that heart tissue has its mechanisms to alleviate inflammation. Elevated levels of brain natriuretic peptide (BNP) have been observed in recipients after heart transplantation, which further increased during acute rejection. *In vitro* co-culture of BNP with peripheral blood MNCs from recipients resulted in upregulated caspase-3 expression and apoptosis in CD8+ T cells together with reduced TNF-α, IL-1α, and IL-6 secretion [73]. The value of BNP as a diagnostic biomarker will be discussed later.

### 3.3 Antibody-mediated Rejection

Currently, plasmapheresis, high-dose intravenous immunoglobulin (IVIg), and rituximab are used to treat AMR in heart transplant recipients [74]; however, these treatments are less efficient in patients with higher DSA levels [75]. Moreover, most cases appear to have mixed rejections where the infiltration of MNCs reduces the efficiency of standard treatments [76]. Accordingly, some studies have targeted T lymphocyte subsets involved in AMR. For instance, thymoglobulin has been shown to prevent AMR by reducing the proportion of T follicular helper cells (Tfh) that play a major role in B cell activation and antibody production. Tfh cells may not be sufficiently suppressed by standard immunosuppressive therapy, including TAC/MMF/steroids. Therefore, thymoglobulin might be helpful in the prevention of AMR, especially in sensitized recipients [77]. Additionally, LFA-1 blockade with anti-LFA-1 mAb reduced IL-21 expression in humanized CD52 transgenic mice, thus the size and number of germinal centers decreased significantly in AMR [78].

The other therapeutic strategy to prevent or treat AMR is complement inhibition. Eculizumab has been used to treat a wide range of antibody-mediated renal disorders, including AMR [79]. In a single-arm, open-label trial 20 patients with panel reactive antibodies (PRA) ≥ 70% and mean fluorescence intensity (MFI) DSA of 6250 received 9 infusions of eculizumab during the first 2 months of transplantation (besides the standard of care), which resulted in a significant reduction in biopsy-proven AMR [80]. In the 5-year follow-up of these patients, there were no cases of pathological AMR2 (pAMR2) and no reports of left ventricular dysfunction. However, there were three deaths, one episode of pAMR1, and one patient with minimal *de novo* cardiac allograft vasculopathy. Accordingly, eculizumab showed a non-significant advantage with a lower incidence of AMR or death compared with the control group [81]. Furthermore, 18-kDa translocator protein (TSPO) ligands, including FGIN1-27 or Ro5-4864, inhibited the proliferation and differentiation of B cells into active plasma cells and reduced antibody secretion *in vitro*. In a mixed-AMR rat model, treatment with FGIN1-27 or Ro5-4864 attenuated DSA-mediated cardiac-allograft injury, prolonged graft survival, and reduced the numbers of graft-infiltrating B cells, T cells, and macrophages. TSPO ligands were hypothesized to inhibit the metabolic activity of B cells by reducing the expression of pyruvate dehydrogenase kinase 1 (PDK-1) and protein complexes I, II, and IV of the electron transport chain [82]. However, the efficacy and safety of TSPO ligands in AMR patients remains to be determined. Finally, monthly ECP improved the clinical symptoms of a DSA-negative patient who was unresponsive to the standard treatment with anti-AT1R and five other autoantibodies [83].

#### 3.3.1 Desensitization

Allosensitization or the production of antibodies against alloantigens is a common complication in heart transplantation, which is associated with prolonged waiting time and poor outcomes. Sensitization might occur due to blood transfusion, pregnancy, previous transplants, or ventricular assist device placement. Currently, there is no consensus on when and how desensitization should be performed [84]; therefore, many desensitization protocols have been proposed. One of the first studies suggested daily tacrolimus and MMF, weekly protein-A Immunoadsorption, IVIg, and daclizumab for highly sensitized heart transplant candidates. Protein-A immunoadsorption was continued during the early post-transplant period, leading to decreased anti-HLA antibody levels and successful transplantation [85]. In another study, the proteasome inhibitor, bortezomib, and plasmapheresis (PP) were added to the combination therapy of IVIg and rituximab in six patients refractory to desensitization, which resulted in four successful cardiac transplants and a significant reduction in cPRA and alloantibody levels. Two other patients died from sepsis, one before and the other early after transplantation [86]. A similar desensitization protocol was used to treat a highly sensitized pediatric case that remained rejection-free during the follow-up period and had no detectable DSA [87]. Later, different doses of bortezomib in combination with a single dose of rituximab and PP were tested in a group of sensitized patients that demonstrated a higher efficacy for high doses in reducing antibody titers. Finally, 19 out of 44 patients were transplanted with a low acute rejection rate [88]. Desensitization of nine patients with repeated cycles of PP, IVIg, and carfilzomib, another proteasome inhibitor, significantly reduced the cPRA values. Six patients were transplanted and survived during the 3-year follow-up. Nonetheless, this study reported thrombocytopenia as a side effect of carfilzomib [89]. Bortezomib could also reduce the level of AT1R antibodies in a group of sensitized patients [90]. Another desensitization protocol, including administration of IVIg and rituximab followed by bortezomib and PP, in ten highly sensitized patients showed efficacy in seven patients who underwent transplantation, but cPRA reduction was not significant in three patients. All patients showed adverse events such as coagulopathy, bone marrow suppression, and infection. One patient experienced clinical rejection and three patients had elevated DSA levels [91]. In addition, in a single-center study, 43 highly sensitized patients underwent desensitization therapy with bortezomib and PP, which resulted in a slight decrease in mean HLA-I and HLA-II cPRA. The patients had comparable one-year survival to the non-sensitized controls but they experienced more AMR episodes and developed more *de novo* DSAs. Moreover, this treatment was associated with an increased risk of thrombocytopenia, infectious complications, and neuropathy [92]. Furthermore, desensitization in 25 sensitized candidates with plasma exchange, IVIg, and bortezomib resulted in a significant reduction in anti-HLA-I titers and HLA-I cPRA but not anti-HLA-II titers. Nineteen patients were transplanted, with a 1-year survival of 89% and an AMR rate of 33% [93]. These findings indicated that despite the relative efficiency of proteasome inhibitors, *i.e.* bortezomib and carfilzomib, in desensitization, adverse events especially bone marrow suppression should be taken into account.

The other desensitization protocol suggested for pediatric patients with PRA >10% included PP, IVIg, cyclophosphamide, and rituximab, which caused a significant reduction of PRA values and comparable survival and rejection rates with non-sensitized patients [94]. In another study, immunoadsorption (IA) was added to the standard desensitization treatments either before or after heart transplantation, resulting in a significant reduction in the percentage of PRA and *de novo* antibody levels [95]. Recently, one *ex vivo* experiment using intraoperative IA as a rapid desensitization method showed a significant reduction in individual allele MFI to below clinically relevant levels with the majority of cases below the lower positive detection limit within 120 min, suggesting the efficacy of intraoperative IA over some time typical for heart transplantation [96]. In addition, one center reported acceptable transplant outcomes with an urgent desensitization protocol, including immunosuppressants (tacrolimus, MMF, and methylprednisolone), preoperative double filtration plasmapheresis (DFPP), rituximab, IVIg, and intraoperative PP during cardiopulmonary bypass if necessary. The first-year survival of these patients was comparable with non-sensitized recipients; however, AMR and ACR episodes were observed in 8% and 16% of patients, respectively [97]. A desensitization protocol including intraoperative tocilizumab, rituximab, and plasma exchange followed by a six-month course of IgA- and IgM-enriched human immunoglobulins (IgGAM) in 19 sensitized patients, which resulted in similar rates of rejection and survival to non-sensitized candidates [98].

Daratumumab, a monoclonal anti-CD38 antibody that targets plasma cells, has also been used to treat a pediatric case resistant to the rituximab, bortezomib, and IVIG treatment. Six weekly doses of daratumumab in combination with MMF, prednisolone, and monthly infusion of IVIg resulted in a stable transplant with no infusion reactions or serious infection [99]. Furthermore, an experimental study targeted memory B cells with cytotoxic T lymphocyte-associated antigen-4Ig (CTLA-4Ig) in mice, which prevented their differentiation into Ab-secreting cells [100]. To evaluate the efficacy of co-stimulation blockade in desensitization, four highly sensitized patients were treated with proteasome inhibitors, dexamethasone and belatacept (CTLA-4Ig). Two patients received plasmapheresis in addition to this protocol. This treatment led to a significant reduction of HLA antibodies and a decrease in the frequency of Tfh and memory B cells. Three patients were transplanted, one of whom developed graft dysfunction due to cellular rejection [101]. However, in a recent study, five highly sensitized patients underwent desensitization therapy with plasma cell depletion therapy (with proteasome inhibitor or daratumumab) and co-stimulation blockade that led to a mean cPRA reduction from 98% to 70% and a non-significant decrease in average MFI values [102]. Regarding inconsistent outcomes, it seems that the efficacy of co-stimulation blockers in desensitization needs further investigation.

A comparison between three groups of patients receiving no induction, induction, and desensitization therapy (PP, IVIg, corticosteroids, and rabbit antithymocyte globulin) before transplantation showed no significant difference in 12-month survival, CAV, and infection rates across groups. Nonetheless, the incidence of acute rejection was lower in the induction and desensitization groups [103]. Extracorporeal membrane oxygenation (ECMO) has also been used for desensitization of cardiac transplant candidates. Urgent desensitization in six patients with PP under ECMO support before transplantation resulted in 100% hospital survival post-transplant, but four out of six patients showed major complications, including hemorrhagic shocks and infectious events. One patient experienced repeated AMR episodes and expired 15 months after transplantation [104].

Nonetheless, it should be noted that the results of desensitization therapy vary from patient to patient and center to center. A comparison between sensitized patients (PRAs > 10%) treated with combination therapy (PP, IVIg, and rituximab), sensitized patients without treatment, and a group of non-sensitized patients demonstrated a similar 5-year survival and freedom from cardiac allograft vasculopathy between groups [105]. In addition, allosensitization and response to treatment might be affected by the genetic background of the recipients. One research showed a significant association between certain polymorphisms of IL-4Rα, IFN-γ, and IL-12 genes and the results of desensitization. Higher serum levels of IL-12 and certain genotypes of this cytokine were correlated with desensitization resistance. This study suggested modifications in PP frequency and IVIg doses in such patients [106]. There are also reports of gene polymorphisms that cause high expression of TNF-α and IL-6 and intermediate expression of TGF-β1 and IL-10 in patients resistant to desensitization [107]. Regarding the role of pregnancy in allosensitization, one investigation demonstrated a higher efficacy of desensitization in male than in female transplant candidates. Therefore, female patients might require more intensive desensitization protocols [108]. Finally, anti-ABO antibodies may decrease with desensitization. In a pediatric case with an ABO-incompatible heart transplant, treatment with rituximab/IVIg resulted in a decreased titer of anti-A isohemagglutinin along with reduced cPRA, leading to successful transplantation [109].

### 3.4 Chronic Rejection

As mentioned earlier, there is no definitive treatment for chronic allograft dysfunction; however, efforts have been made to prevent or attenuate its progression. One cohort study comparing the efficacy of low-dose-tacrolimus/sirolimus or full-dose-tacrolimus/MMF in heart transplant recipients showed no superiority of sirolimus to full-dose tacrolimus therapy in preventing CAV. After eight years of follow-up, CAV severity, acute rejection rate, creatinine levels, and survival were comparable between the two groups [110]. A retrospective study of 61 pediatric heart transplant recipients showed that treatment with everolimus started in the first 14 days post-transplant precluded the development of CAV over a median follow-up of 4.3 years; however, adverse effects such as lymphoproliferative disorders and renal failure were observed in patients [111]. Therefore, it appears that novel approaches are required for the management of CAV in patients; accordingly, there are several experimental studies offering alternatives for the prevention or treatment of CAV. For instance, perfusion of a donor mouse heart with MMF-loaded PEG-PLGA nanoparticles for direct delivery and sustained release of MMF before transplantation ameliorated CAV by suppressing intragraft proinflammatory cytokines and chemokines [112]. Furthermore, combination therapy of tacrolimus and AS2553627, a JAK inhibitor, for 90 days significantly attenuated CAV and fibrosis in a rat model of cardiac transplant [113]. Metformin has also shown protective effects against CAV in a murine model by limiting ischemia-reperfusion injury probably *via* activation of AMP-activated kinase (AMPK) [114]. The other substance suggested to mitigate CAV was adiponectin (APN), an adipose tissue-specific secretory protein. APN-sense transgenic mice showed significantly lower mRNA levels of IFN-γ, TNF-α, IL-2, IL-6, and MCP-1 after cardiac transplantation. Proliferation of smooth muscle cells stimulated by activated T cells was suppressed with APN, but this effect was neutralized by AMPK inhibition [115]. Treatment of a mice model of chronic allograft rejection with anti-interleukin-12/23p40 antibody resulted in reduced levels of intragraft IL-6, IFN-γ, IL-17a, CCL2, and CCL20 [116]. Moreover, administration of D-4F, an apolipoprotein-A-I mimetic peptide with anti-inflammatory/antioxidant properties, to a murine model of CAV alleviated the severity of intimal lesions. It also reduced CD4+, CD8+, and CXCR3+ T cell infiltration in graft and increased the expression of heme oxygenase-1 [117]. Considering the elevated serum levels of Leukotriene B4 in humans and the experimental model of CAV, Bestatin, an LTB4 inhibitor, was administered to the mice, which mitigated neointimal hyperplasia in allograft [118]. Arteries express Ephrin-B2 molecules that bind to EphB4 on circulating monocytes, facilitating endothelial adhesion. Hence, the EphB4 monomer was used to inhibit Ephrin-B2-EphB4 binding in an aortic transplant model. Rats treated with EphB4 monomer had fewer macrophages and T cells in the aortic allografts at 28 days, as well as significantly less neointima formation, suggesting Ephin-B2-EphB4 axis as a target for prevention or treatment of allograft vasculopathy [119].

Considering the role of DSA and B-lymphocytes in the initiation and progression of CAV, B cells have been targeted to prevent and treat chronic allograft dysfunction. Administration of FX1, a B cell lymphoma 6 (Bcl6) inhibitor, prolonged survival of cardiac graft and prevented vascular fibrosis and occlusion in a mice model of chronic cardiac rejection. FX1 decreased the number of splenic plasma cells, germinal center B cells, IgG1+ B cells, and Tfh cells, as well as DSA titers [120]. Similarly, the B lymphocyte stimulator (BLyS) inhibitor, belimumab, prevented B-cell activation and differentiation, prolonged allograft survival, and attenuated chronic rejection. Belimumab significantly inhibited interstitial fibrosis and reduced DSA levels and C4d deposition. Inflammatory cell infiltration, frequencies of B cells, plasma cells, and IgG-producing cells in mice spleen, lymph nodes, bone marrow, and blood circulation were also reduced [121]. In addition, BTK signaling blockade using the BTK inhibitor, Ibrutinib, abrogated B cell activation, differentiation, and antibody production, thus alleviating neointimal hyperplasia, interstitial fibrosis, inflammatory cell infiltration, and C4d deposition [122]. Additionally, injection of adenovirus-IL-10-treated macrophages into the mice reduced perivascular fibrosis, apoptosis, and inflammation, mitigating chronic rejection of heart transplant [123]. Finally, autologous hematopoietic stem cell transplantation (HSCT) improved cardiac allograft survival in mice, which was associated with increased intragraft Foxp3+ Treg cells, reduced intragraft B cells, and decreased serum DSA levels [124].

## 4 ADVANCES IN DIAGNOSTIC BIOMARKERS

Several biomarkers have already been suggested to replace invasive methods such as EMB for diagnosing heart transplant rejection. In addition to the safety of biomarker tests, the rapidity and low cost make them an appealing alternative for regular monitoring of post-transplant complications. However, their specificity, reproducibility, and integration into clinical workflows require further investigations. Herein, a summary of the biomarkers proposed for early diagnosis of heart transplant rejection has been provided (Table **1**).

B-type natriuretic peptide (BNP), a marker of ventricular wall stress, was one of the first biomarkers introduced for the diagnosis of allograft dysfunction and cardiac allograft vasculopathy. A mean 5-year follow-up of heart transplant recipients suggested elevated levels of BNP as a significant predictor of cardiovascular death [125]. N-terminal probrain natriuretic peptide (NT-proBNP) and CRP, as markers of congestive heart failure, in combination have shown predictive value for the development of CAV and all-cause mortality after transplantation [126]. Elevated serum levels of NT-proBNP alone might be suggestive of acute rejection of cardiac allograft [127] or ischemia-reperfusion injury [128]. The other biomarker for diagnosing acute rejection of cardiac transplant is soluble Suppression of Tumorigenicity 2 protein (sST2), an IL-1 receptor family member, and its upregulation was associated with pathological findings of ACR; the predictive value of sST2 improved when combined with NT-proBNP levels [129]. Additionally, a long-term follow-up of 198 heart transplant recipients showed that NT-proBNP plasma concentration directly before transplant, plasma concentration of fibrinogen one month after transplant, and diabetes were independent predictors of CAV [130]. Moreover, one pediatric study demonstrated a significant association between acute rejection episodes and elevated concentration of NT-proBNP and cardiac troponin T (cTnT) independently. After treatment, cTnT levels were reduced paralleled with EMB results improvement [131]. Another study showed that cardiac troponin I (cTnI) level was significantly higher in rejection *versus* non-rejection serum samples and increased in a graded manner with biopsy scores [132]. Elevated high-sensitivity troponin T (hsTnT) values during posttransplant intensive care admission and prolonged coronary bypass time have also been shown to predict primary graft dysfunction [133]. Another study revealed a significant association between hsTnT at 48 h after transplantation and mortality and a negative correlation between hsTnT levels and days alive and out of hospital [134]. Similarly, a cohort study of 156 patients for approximately 9 years after transplantation demonstrated a positive correlation between high-sensitivity troponin I (hsTnI) levels and CAV grade after adjustment for age, sex, BMI, hypertension, diabetes, hyperlipidemia, estimated glomerular filtration rate, and history of ACR. In addition, hsTnI levels above the median value were suggested as predictors of retransplantation or death [135]. Although BNP and troponin appear to be specific to heart tissue and sensitive to subclinical damages, their utility as diagnostic biomarkers for acute or chronic rejection is yet to be confirmed since the serum level of BNP and troponin might increase in any other injurious condition to heart tissue. One meta-analysis including 12 studies with 993 patients and 3,803 EMBs, most of which paired with cTn levels, showed insufficient specificity of cTn for diagnosis of acute rejection; nonetheless, hs-cTn assays might have an acceptable sensitivity and a negative predictive value to exclude acute rejection [136].

As mentioned before, ST2 combined with NT-proBNP has been proposed as a diagnostic biomarker for ACR [129]. In addition, one prospective study reported an increased incidence of AMR in heart transplant recipients with ST2 levels higher than 35 ng/mL post-transplant [137]. In line with this, a pediatric study showed that at times of biopsy-diagnosed cellular and/or antibody-mediated rejection, serum ST2 was elevated compared to rejection-free time points. This research suggested TNF-α and IL-1β as upstream activators of ST2 expression [138]. Besides, there are studies proposing the plasma concentrations of SERCA2a involved in cardiac homeostasis to discriminate between patients with and without PGD [139] and higher serum levels of IL-4,-6,-10,-21,-23,-31,-33, TNF-α, and soluble activation marker CD40 ligand in CAV patients [140].

MicroRNAs (miR)s are small noncoding RNA molecules that regulate the posttranscriptional expression of target genes. MiR expression studies on experimental heart transplant models showed an increased expression of miR-182 and miR-155 in rejecting cardiac allograft, mononuclear cell infiltrates, peripheral blood mononuclear cells, and plasma compared to syngeneic transplants [141, 142]. In addition, increased cardiac miR-21 levels were observed in both human and experimental models with chronic allograft rejection. MiR-21 was induced upon treatment with IL-6 in a monocyte cell line and promoted monocyte transition to fibrocytes [143]. Likewise, seven miRs have been demonstrated to differentially express between normal and rejecting heart allografts, including miR-10a, miR-21, miR-31, miR-92a, miR-142-3p miR-155, and miR-451. Moreover, miR-10a, miR-31, miR-92a, and miR-155 showed different serological expression in patients with allograft rejection [144]. In patients diagnosed with CAV, plasma levels of miR-628-5p and miR-155 were significantly increased. Of note, a miR628-5p value above 1.336 was able to predict CAV with considerable sensitivity and specificity [145]. It was also observed that miR-326 and miR-155 expression levels increased after 24h and 72h of surgery in the rejection group compared to the non-rejection patients, but this was not statistically significant. However, cTnT levels in rejection patients increased significantly [146]. Analysis of miR expression in the cardiac biopsies suggested elevated levels of miRs 208a, 126-5p, and 135a-5p for diagnosis of mixed rejection, miRs 27b-3p, 29b-3p, and 199a-3p for acute rejection, and a combination of miRs 208a, 29b-3p, 135a-5p, and 144-3p for AMR. Notably, miR 126-5p was expressed in endothelial cells during acute rejection and in cardiomyocytes during AMR [147]. Furthermore, one next-generation small RNA sequencing study found three miRs with significantly increased serum levels in patients with biopsy-proven cardiac rejection compared to patients without rejection, including miR-139-5p, miR-151a-5p, and miR-186-5p. Besides, miR-29c-3p was upregulated in ACR, while miR-486-5p was a potential biomarker of AMR [148]. In addition, serum miR-144-3p was found to discriminate between Grade ≥2R ACR and low-grade or non-rejection status [149]. MiR-652-3p overexpression has been suggested to discriminate between patients with and without ACR. Moreover, the combined serum level of miR-144-3p and miR-652-3p was significantly higher in patients with rejection [150]. HEARTBiT, a transcriptomic signature including 9 mRNA transcripts associated with acute cellular rejection, showed 47% specificity and more than 90% sensitivity for excluding ACR in adult heart allograft patients [151]. Later, a CD4+ T cell gene signature (TGS) panel that tracks conventional T cells (Tconv) and Treg cells in adult heart transplant recipients showed decreased Treg- and increased Tconv-gene expression in rejection compared with non-rejection samples. The TGS panel was able to discriminate between acute rejection and non-rejection samples and, when combined with HEARTBiT, showed higher specificity than either model alone [152].

Circulating donor-derived cell-free DNA (dd-cfDNA), released by damaged cells of allografts, is the other genetic product introduced to predict allograft rejection after heart transplantation since it has shown a significant correlation with EMB results [153]. Evaluation of 740 heart transplant recipients from 26 centers and a single-center cohort of 33 patients demonstrated plasma dd-cfDNA as a good negative predictor for patients at risk of AMR as dd-cfDNA levels were elevated 3-fold in AMR compared with patients without AMR [154]. One pediatric study reported a dd-cfDNA peak within the first 2 days of transplantation that declined to baseline by 6-9 days. However, mortality was associated with elevated levels, late rise, or slow decline of dd-cfDNA in the first week [155]. Similarly, a cohort study showed high plasma levels of dd-cfDNA immediately after reperfusion that declined within 10 days in stable patients but remained high in biopsy-proven rejection grades 1R and 2R. Notably, dd-cfDNA significantly increased 9-30 days before biopsy-proven rejection episodes [156]. In addition, a clinical study including 171 patients with a median follow-up of 17.7 months showed that dd-cfDNA percentages declined to 0.13% after surgery within 4 weeks. Nonetheless, dd-cfDNA increased in acute rejection with a negative predictive value of 99% for rejection and exhibited a positive correlation with rejection grades. This rise was detected weeks before EMB diagnosis of rejection. Moreover, the dd-cfDNA percentage showed discriminative potential between AMR and ACR [157]. Furthermore, a prospective study reported that total cell-free DNA (TCF) and dd-cfDNA levels were associated with EMB results and clinical outcomes. Higher TCF values were correlated with increased risk of mortality and infection, whereas increased dd-cfDNA was associated with rejection and CAV [158]. Accordingly, one center replaced EMB surveillance with dd-cfDNA monitoring. During six months, dd-cfDNA remained low in most patients but those with values more than 0.25% were subjected to EMB. Of note, there was no missed rejection case [159]. The combination of dd-cfDNA and gene expression profiling (GEP) data also reduced the need for biopsy during the first year of heart transplantation, offering an alternative for EMB surveillance [160]. In addition, the levels of circulating mitochondrial genes, *e.g.* mitochondrial Ca^2+^ uniporter complex, have been demonstrated to discriminate between rejection and non-rejection status in heart transplant recipients. One hundred and eleven mitochondrial genes have been identified in the serum of patients, of which 28 were differentially expressed in patients with acute rejection [161]. Despite findings of numerous studies offering miRs, dd-cfDNA, and gene expression profiles to discriminate between rejection and non-rejection status, the applicability of these results in practice faces challenges; for instance, the results of the experimental study could not be easily translated to human patients unless confirmed with clinical studies. Moreover, the miRs that have been studied in biopsy samples do not eliminate the need for invasive diagnostic procedures. In addition, dd-cfDNA and miR expression evaluation requires costly equipment and highly trained staff that could not be provided in every transplant center.

Exosomes are cell-derived circulating vesicles that play an important role in cell-cell communication. In spite of being expensive and challenging, analysis of exosomal protein content may be considered for monitoring rejection in transplant recipients. The analysis of serum exosomes from non-rejection recipients and ACR and AMR patients revealed 15 differentially expressed proteins across groups. Eight out of 15 proteins were involved in the immune response, in particular, complement activation, adaptive immunity, and coagulation [162]. Furthermore, the plasma exosome analysis in four patients showed donor troponin protein and mRNA expression at all follow-up time points. One patient developed AMR on the 14^th^ day of EMB with concurrent detection of C4d in donor heart exosomes that resolved with treatment [163]. Analysis of extracellular vesicle (EV) concentration from 90 patients showed a remarkable increase in the number and a decrease in the diameter of EVs in the patients diagnosed with rejection. This was observed in both ACR and AMR; however, surface markers differed between the two groups as CD3, CD2, ROR1, SSEA-4, HLA-I, and CD41b discriminated between ACR and stable recipients, whereas HLA-II, CD326, CD19, CD25, CD20, ROR1, SSEA-4, HLA-I, and CD41b were prominent in patients with AMR [164]. Recently, donor-reactive T cell small extracellular vesicle (sEV) profiling in experimental models and heart transplant recipients showed time-specific expression of alloreactive and regulatory markers, indicating early acute cellular rejection. Moreover, the sEVs collected from recipients showed targeted cytotoxicity to donor cardiomyocytes presented by enhanced apoptosis [165].

RanGTPase AP1 (RANGAP1), involved in nucleocytoplasmic transport, has also been proposed as a diagnostic biomarker since elevated serum levels of RANGAP1 and NT-proBNP differentiated between ACR and non-ACR status independently [166]. Finally, immunoPET, utilizing antibodies conjugated to radioisotopes, has shown the potential to detect graft rejection by visualizing the dynamic distribution of immune cells in the body. In an experiment, OX40 immunoPET illustrated alloreactive T cells in the allograft and draining lymph node, which was absent in the isograft tissues [167]. Regarding the risk of exposure to radioisotopes, the safety of such diagnostic methods might be doubted for regular monitoring of patients.

## CONCLUSION

Despite advances in surgical techniques, post-transplant care, immunosuppressive agents, and monitoring tests, allograft rejection, particularly CAV, is considered a major problem in achieving successful heart transplantation. Recent findings about the implication of certain cells, cytokines, and signaling molecules in developing acute and/or chronic rejection suggest new therapeutic targets to prevent and treat alloimmune injury. Furthermore, certain cell products and proinflammatory molecules, as well as genetic biomarkers, *e.g.* miRNAs and dd-cfDNA, have been shown to have diagnostic or prognostic value for different types of allograft rejection. These biomarkers may facilitate the diagnosis of alloimmune responses by discovering early modifications in immune cell differentiation and functio, or by detecting subclinical graft injury. Considering the risk of EMB, which is currently the gold standard for diagnosis of rejection, establishing non-invasive and cost-effective biomarker tests can be helpful in safe allograft surveillance. Nonetheless, the feasibility, sensitivity, and specificity of proposed biomarkers need further evaluation.

## Figures and Tables

**Table 1:** Biomarkers for diagnosis of cardiac transplant rejection.

Biomarker	PGD	ACR	AMR	CAV	Mortality	References
BNP NT-proBNP		●		● ●	● ●	[125-127, 129-131, 166]
sST2		●	●			[129, 137, 138]
cTnT cTnI hsTnT hsTnI	●	● ●		●	● ●	[131-134, 137, 146]
SERCA2a	●					[139]
miR-182 and miR-155		● (mice)				[141, 142]
miR-21				● (mice and human)		[143]
miR-10a, miR-21, miR-31, miR-92a, miR-142-3p miR-155, and miR-451		●				[144]
miR-628-5p and miR-155				●		[145]
miR-326 and miR-155		●				[146]
miRs 27b-3p, 29b-3p, and 199a-3p		●				[147]
miRs 208a, 29b-3p, 135a-5p, and 144-3p			●			[147]
miR-139-5p, miR-151a-5p, miR-186-5p, miR-29c-3p, miR-486-5p		●				[148]
miR-486-5p			●			[148]
miR-144-3p		●				[149]
miR-144-3p and miR-652-3p		●				[150]
dd-cfDNA		●	●	●	●	[153, 154, 156-160]
Circulating mitochondrial genes		●				[161]
Exosomal proteins		●	●			[162]
C4d in exosomes			●			[163]
Surface markers of extracellular vesicles		●	●			[164]
T cell small extracellular vesicle		● (mice and human)				[165]
RANGAP1		●				[166]
OX40 (immunoPET)		● (mice)				[167]

**Abbreviations:** PGD: primary graft dysfunction, ACR: acute cellular rejection, AMR: acute antibody-mediated rejection, CAV: chronic allograft vasculopathy, BNP: brain natriuretic peptide, NT-proBNP: N-terminal probrain natriuretic peptide, sST2: soluble Suppression of Tumorigenicity 2 protein, cTnT: cardiac troponin T, cTnI: cardiac troponin I, hsTnT: High-sensitivity troponin T, hsTnI: High-sensitivity troponin I, SERCA2a: sarcoplasmic/endoplasmic reticulum Ca^2+^-ATPase, miR: microRNA, dd-cfDNA: Donor-derived cell-free DNA, RANGAP1: Ran GTPase-activating protein 1.

## LIST OF ABBREVIATIONS

ACR: Acute Cellular Rejection
AMPK: AMP-activated Kinase
AMR: Acute Antibody-mediated Rejection
AT1R: Angiotensin II Receptor type 1
Bcl-2: B-cell Lymphoma 2
BD: Brain-dead
BNP: Brain Natriuretic Peptide
BTK: Bruton's Tyrosine Kinase
CAV: Chronic Allograft Vasculopathy
CCL: Chemokine (C-C motif) Ligand
CMV: Cytomegalovirus
CRP: C-reactive Protein
CTLA-4Ig: Cytotoxic T Lymphocyte-associated Antigen-4Ig
cTnI: cardiac Troponin I
cTnT: cardiac Troponin T
CXCL: Chemokine (C-X-C motif) Ligand
DAMP: Danger-associated Molecular Patterns
dd-cfDNA: Donor-derived cell-free DNA
DFPP: Double Filtration Plasmapheresis
DSA: Donor-specific Antibodies
EBV: Epstein-barr Virus
ECMO: Extracorporeal Membrane Oxygenation
EMB: Endomyocardial Biopsy
eNOS: Endothelial Nitric Oxide Synthase
FcγR: Fc-gamma Receptor
HIF1A: Hypoxia-inducible Factor 1 Subunit Alpha
HLA: Human Leukocyte Antigen
HMGB1: High-mobility Group Box 1
HO-1: Heme Oxygenase-1
hsTnI: High-sensitivity Troponin I
hsTnT: High-sensitivity Troponin T
ICAM-1: Intercellular Adhesion Molecule-1
IFN-γ: Interferon-γ
IgGAM: IgA- and IgM-enriched human immunoglobulins
IL: Interleukin
JAK: Janus Kinase
LNR: Lymphocyte to Neutrophil Ratio
MCP-1: Monocyte Chemoattractant Protein-1
MICA: MHC class I chain-related gene A
miR: microRNA
MMP: Matrix Metalloproteinase
MNC: Mononuclear Cell
MP: Methylprednisolone
MPO: Myeloperoxidase
MSC: Mesenchymal Stem Cells
Nr4A1: Nuclear receptor subfamily 4 Group A member 1
NT-proBNP: N-terminal Probrain Natriuretic Peptide
P2X7R: Purinergic Receptor-7
PDK-1: Pyruvate Dehydrogenase Kinase 1
PGD: Primary Graft Dysfunction
PP: plasmapheresis
PRA: Panel Reactive Antibodies
RANGAP1: Ran GTPase-activating Protein 1
SERCA2a: Sarcoplasmic/Endoplasmic Reticulum Ca^2+^-ATPase
sST2: soluble Suppression of Tumorigenicity 2 protein
T3: Tri-iodothyronine
TCR: T-cell Receptor
TGF-β: Transforming Growth Factor-beta
TLR4: Toll-like Receptor 4
TNF-α: Tumor Necrosis Factor-alpha
TSPO: Translocator Protein
VCAM-1: Vascular Adhesion Molecule-1
